# The Application of 3D Anatomy for Teaching Veterinary Clinical Neurology

**DOI:** 10.3390/ani13101601

**Published:** 2023-05-10

**Authors:** Lidia Blázquez-Llorca, Lubna Morales de Paz, Rosario Martín-Orti, Inmaculada Santos-Álvarez, María E. Fernández-Valle, David Castejón, María I. García-Real, Raquel Salgüero-Fernández, Pilar Pérez-Lloret, Nerea Moreno, Sara Jiménez, María J. Herrero-Fernández, Juncal González-Soriano

**Affiliations:** 1 Departamento de Anatomía y Embriología, Sección Departamental de Anatomía y Embriología (Veterinaria), Facultad de Veterinaria, Universidad Complutense de Madrid, Avenida Puerta de Hierro s/n, 28040 Madrid, Spain; lidiblaz@ucm.es (L.B.-L.); lubnamor@ucm.es (L.M.d.P.); rosamart@ucm.es (R.M.-O.); inmasant@ucm.es (I.S.-Á.); pilper01@ucm.es (P.P.-L.); 2 ICTS Bioimagen Complutense, Universidad Complutense de Madrid, Paseo de Juan XXIII 1, 28040 Madrid, Spain; evalle@ucm.es (M.E.F.-V.); dcastejon@ucm.es (D.C.); 3 Departamento de Medicina y Cirugía, Facultad de Veterinaria, Universidad Complutense de Madrid, Avenida Puerta de Hierro s/n, 28040 Madrid, Spain; isagreal@vet.ucm.es (M.I.G.-R.); rsalgu01@ucm.es (R.S.-F.); 4 Hospital Veterinario Veterios, Calle Arrastaria, 23, 28022 Madrid, Spain; 5 Departamento de Biología Celular, Facultad de Biología, Universidad Complutense de Madrid, Avenida José Antonio Novais 12, 28040 Madrid, Spain; nerea@bio.ucm.es (N.M.); sajime01@ucm.es (S.J.); 6 Departamento de Mineralogía y Petrología, Facultad de Geología, Universidad Complutense, Avenida José Antonio Novais 12, 28040 Madrid, Spain; mjherrer@ucm.es

**Keywords:** anatomy, neuroanatomy, teaching, magnetic resonance imaging, segmentation

## Abstract

**Simple Summary:**

Anatomy is regarded as a key element in medical and veterinary education all over the world. It is a subject of voluminous and sometimes complex content, which normally causes students’ different difficulties. These difficulties increase dramatically when it comes to the study of neuroanatomy. Hence, there have been continued efforts in developing new methods of teaching, learning and assessment that are aimed at the long-term retention of anatomical and neuroanatomical knowledge. Additionally, clinical neurology can be difficult for veterinary students to comprehend, and there is no doubt that part of its understanding is directly related to the knowledge of neuroanatomy. Therefore, the aim of this present work is to be a teaching tool oriented towards clinical neurology and clinical practice, through 3D reconstructions of magnetic resonance images of normal brains and clinical neurological cases.

**Abstract:**

Neuroanatomy is always a challenging topic for veterinary students. It is widely accepted that understanding the anatomy of the central nervous system (CNS) is essential to explain many of the pathological processes that affect the brain. Although its study has varied over time to achieve this goal, in human and veterinary medicine it is difficult to find a teaching method that associates normal anatomy with pathological alterations of the brain. For the first time, we have created an educational tool that combines neuroanatomy and neuropathology, using different magnetic resonance (MR) images as a basis and EspINA software as analyzer, to obtain segmented structures and 3D reconstructions of the dog brain. We demonstrate that this combination is an optimal tool to help anatomists to understand the encephalon, and additionally to help clinicians to recognize illness including a multitude of neurological problems. In addition, we have tried to see whether photogrammetry, which is a common technique in other sciences, for example geology, could be useful to teach veterinary neuroanatomy. Although we still need further investigations, we have been able to generate 3D reconstructions of the whole brain, with very promising results to date.

## 1. Introduction

Anatomy is a science concerned with the identification and description of the body structures of living things [1]. It focuses on the description of form, or how body structures at different levels look, including the cells, tissues and organs and how they are organized. Consequently, veterinary anatomy is a science of which the main target is the study of animals’ structure and organization.

Neuroanatomy is a very challenging topic for veterinary students, as the main goal is to associate gross anatomy with neurologic functions and, as a second step, to be able to localize neural lesions in the case of neurologic disease [2,3]. In this sense, the teaching of anatomy has evolved to the point of using different innovative methods oriented towards clinical practice [4,5]. In this present paper, we focus on the study of the central nervous system, taking the dog as a model.

Cadaver dissection started in 300 BC, and by the 15th century, it was considered the main tool for studying the structural details of the different bodily organs, including the brain [6,7]. Now, although veterinary professionals, surgeons, anatomy teachers and researchers in the anatomical field cannot completely avoid its use, anatomists often use additional methods [8,9]. In the case of the SNC, the use of different techniques for vascular casting [10] or the plastination of brain specimens is common [11]. Thus, the teaching of the CNS anatomy has evolved to introduce the use of other applied technologies. Among them, imaging techniques, X-rays, computed tomography (also called a CAT scan) or magnetic resonance imaging (MRI), stand out without a doubt. Of course, their use implies that educators must first understand how students mentally manipulate anatomical images [12,13,14]. At this point, it is interesting to remark that the use of three-dimensional reconstructions (3D reconstructions) is an additional and important aid to help veterinary students to study the brain, its morphology and function [2,3,15,16,17]. In fact, 3D reconstructions are commonly used as a teaching tool in other sciences, even those unrelated to veterinary medicine or anatomy, such as geology, for example. Between these methods, photogrammetry is very popular between earth sciences degrees, as it is able to facilitate the characterization of geological materials by the generation of 3D models [18,19]. Unfortunately, there is no previous information on the use of photogrammetry or other similar techniques in biological or medical sciences.

MRI is considered the gold standard for the diagnosis of neural pathology in medical and veterinary neurology [13]. Hence, a thorough understanding of MRI brain anatomy is essential for clinicians and surgeons. In this present paper, we analyzed different normal and pathological MR images of dog brains, by the means of EspINA Interactive Neuron Software 2.8.2 (https://cajalbbp.es/espina/, accessed on 2 May 2023). It is important to remark that EspINA allows the visualization and segmentation of large image stacks datasets, from either electron microscopy or confocal laser microscopy. Thus, the main goal of this present paper is to ascertain, for the first time, whether EspINA 3D reconstructions, proceeding from different quality MR images, could be a useful tool not only for anatomy students, but also to help clinicians recognize illness including a multitude of neurological problems. In addition, we have investigated whether photogrammetry could also be useful to produce 3D reconstructions of the brain, either from biological specimens or from brain sections or MR images.

## 2. Materials and Methods

### 2.1. Material

No dogs were sacrificed to carry out this present study. For the ex vivo experiments, we used four fixed brains, selected from the collection of specimens that are normally used to teach Neuroanatomy in the Department of Anatomy and Embryology (Veterinary Faculty, Complutense University of Madrid). In this case, the MRIs were performed at the Bioimaging Center of the Complutense University of Madrid using a Biospec BMT 47/40 system (Bruker Biospin GmbH, Ettligen, Germany) operating at 4.7 T and equipped with a 12 cm gradient system.

The in vivo MR images were performed with different equipment: a close high field MRI system (Vantage Elan 1.5 T, Canon Medical System, Otawara, Japan) from Hospital Veterios in Madrid and an open C-shape low field MRI system (Panorama 0,23 T Philips Medical Systems, Amsterdam, The Netherlands) located in the Complutense University Veterinary Teaching Hospital. The animals were 4 in total, with 2 in each system: a 9 year old female French bulldog (dog 1) and a 6 year old, neutered female (dog 2), in the first case and a middle-aged female crossbreed dog (dog 3) and a 5 year old French bulldog (dog 4), in the second. The crossbreed animals had a normal neurological examination and no signs of neural disease, whereas the other two (both French bulldogs) showed neurological symptoms.

All images were analyzed and segmented by means of the EspINA software (EspINA Interactive Neuron Analyzer, 2.8.2; https://cajalbbp.es/espina/, accessed on 2 May 2023). The 3D Viewer plugin of Image J (ImageJ 1.53, NIH, Bethesda, MD, USA) was used to generate the 3D view of the surface of the brain from the MRI stack of images of the fixed dog brain.

### 2.2. Methods

Two of the fixed brains were thick-sectioned (2–3 mm) by using a scalpel or simply a knife. After this, the slices were labelled by means of the Mulligan’s staining [20], mainly to identify the cerebral grey matter.

The ex vivo dog brains were drained, immersed in a proton-free susceptibility-matching fluid, Fluorinert FC-40 (Sigma-Aldrich, Saint Louis, MO, USA) and placed inside a 7 cm volume radiofrequency coil. The 3D MRI experiment consisted of a T2-weighted (T2W) Fast spin echo (FSE) sequence with the following parameters: repetition time (TR) = 2500 ms; effective echo time (TE) = 35 ms; number of averaged experiments (NAQ) = 8; field of view (FOV) = 90 × 55 × 55 mm^3^; acquisition matrix size = 448 × 192 × 192. The acquired data were zero-filled to obtain a reconstructed matrix size of 512 × 256 × 256; thus, the spatial resolution was 0.176 × 0.215 × 0.215 mm^3^.

Concerning the in vivo experiments in the 1.5 T MRI system, a 16-channel flex medium coil, flexible 24 × 40 wrap, was used in both dogs (dog 1 and dog 2). A complete brain study was performed; however, the sequence used for this present study was a T2W FSE in a transverse plane, and the technical parameters included the following: TR = 16,569 ms and 14,052 ms, and TE = 105 ms; flip angle 90°/120°; NAQ = 2.0; FOV = 14 × 13 cm; matrix size = 256 × 272; slice thickness = 0.5 mm × 0.5 mm, with a pixel size of 1 mm.

In respect to the low field (0.23 T), an extremity coil was used for the examination of the 2 patients (dog 3 and dog 4). The sequence selected for segmentation in the case of dog 3 was a T2*-weighted (T2*W) fast field echo (FFE) 3D sequence in the transverse plane. The parameters selected for this dog were as follows: TR = 60 ms; TE = 16 ms; NAQ = 1; FOV = 330 × 200 mm; acquisition matrix size = 300 × 200. The slice thickness = 3.5 mm. The spacing between slices = 2.3 mm. The sequence used for the segmentation of dog 4 was a T2W turbo spin echo sequence in the transverse plane, and the parameters selected for this patient were as follows: TR = 4500 ms; TE = 96 ms; NAQ = 6; FOV = 343 × 200 mm; acquisition matrix size = 216 × 172. The slice thickness = 3.0 mm. The spacing between slices = 3.3 mm. In both cases, a complete brain MRI was performed. All in vivo experiments were performed under general anesthesia, and patients were positioned in sternal recumbency.

The segmentation with EspINA was manual as, to date, there are no automatic algorithms to adequately represent the structures of interest. Thus, we segmented all MRI stacks of images on a slice-by-slice basis, sometimes altering the MRI dataset contrast to help the visual identification of structures. The stacks of images of the MRI were analyzed using the EspINA software (Espina Interactive Neuron Analyzer, 2.9.15; Madrid, Spain; https://cajalbbp.es/espina/, accessed on 2 May 2023). Since EspINA allows navigation through the stack of images, it was possible to manually segment the selected brain structures in each single section where they were present. The user can create color-coded categories. The segmentations of a specific brain structure can be included in each category. In addition, the EspINA software has a tool that allows the visualization in 3-dimensions of the reconstructed brain structures. In this present paper, we segmented and 3D reconstructed the following structures from MR images: the caudate nucleus, the cerebellum, the cerebral peduncles, the colliculi, the hippocampus, the lateral ventricles, the pyramids, the mesencephalon, the neocortex, the periaqueductal grey matter, the thalamus and the third ventricle,

Photogrammetry is the science of collecting information from 2D photos. By combining enough overlapping images of the same features, it is possible to generate realistic 3D representations of topographic surfaces. That is the reason why we tried to use photogrammetry to 3D reconstruct the brain surface. In this process, there are four main steps: (1) acquisition of specimen’s photographs; (2) calculation of the camera position from some referenced points; (3) generation of a point cloud; (4) processing of the point cloud.

**Figure 1:**
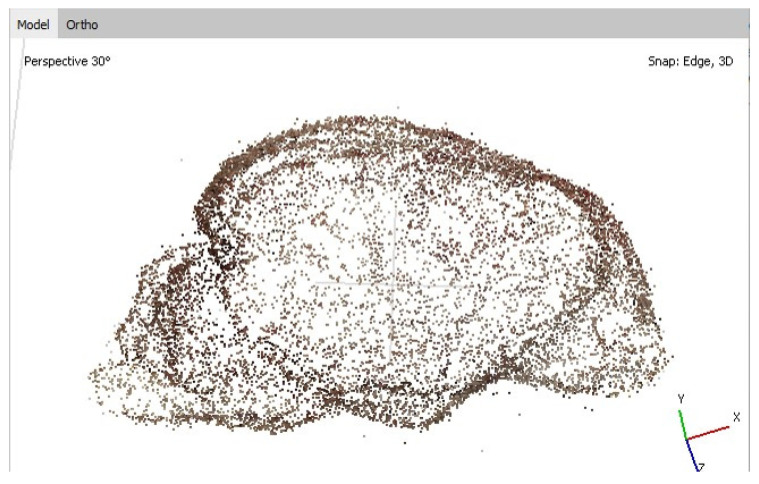
Example of point cloud obtained by the overlapping of a number of photographs.

The model is based on photographs taken from different angles to record the total contour of the object, with a minimum overlapping (30%) of the images. The optimal lighting is a natural and indirect light, so that shaded areas are avoided. The photographs are then included in the AGISOFT Photoscan Professional Software environment 1.1 (AGISOFT PhotoScan LLC, 2014, Saint Petersburg, Russia), with the correspondent methodology [21]. After this processing, the overlapped photographs generate a point cloud. In our case, we used a total number of 32 photographs, which produced the correspondent point cloud in approximately 25 min, obtaining a mesh with 9944 vertex and 18,741 faces (Figure 1).

An additional step is to include several control points into the model with x, y and z values to establish the model size. Further, as the points’ coordinates and the different camera positions are known, it is possible to create a color texture map by projecting the original photographs onto the mesh surface. Finally, the resulting 3D model is exported as an OBJ file.

## 3. Results

**Figure 2:**
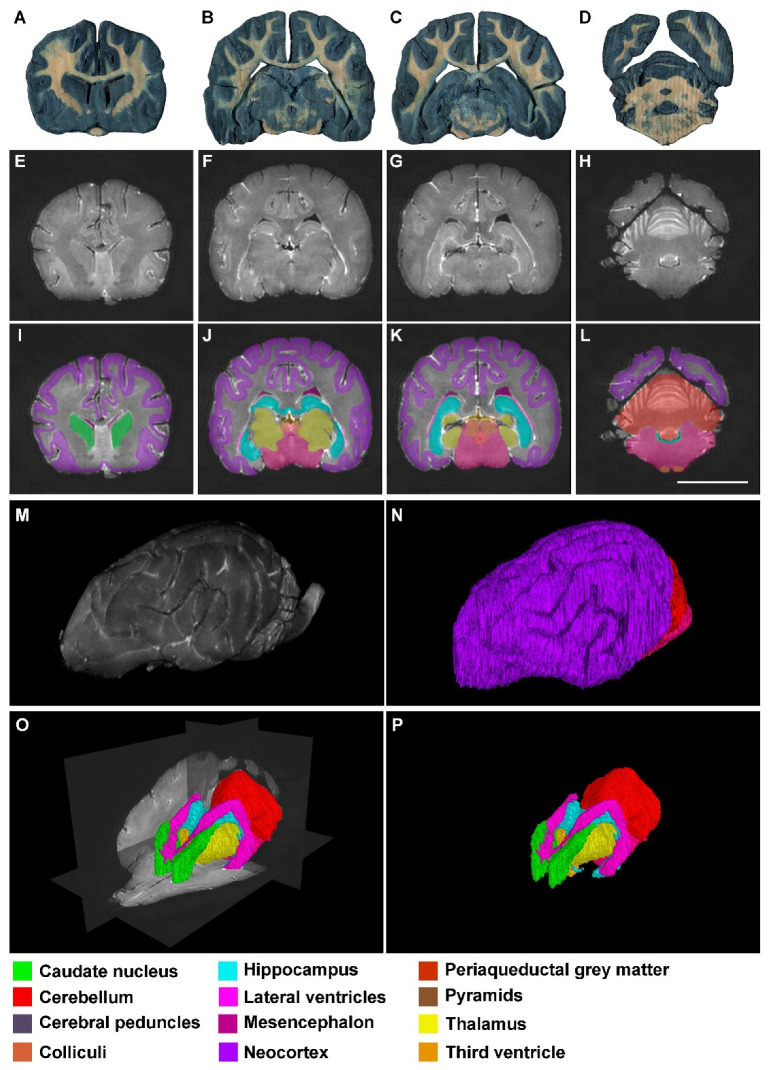
Images of dog brains without any signs of neural disease: (**A**–**D**) thick transversal sections (0.3 mm) stained with Mulligan’s solution. The levels are approximately equivalent to that shown in (**E**–**H**) and (**I**–**L**); (**E**–**H**) Ex vivo transversal MR images (4.7 T) in the anterior–posterior axis; (**I**–**L**) Segmented structures with EspINA analyzer (see the color legend at the bottom of the figure); (**M**) 3D reconstructed brain with ImageJ software, taking the same stack of MR images as the basis; (**N**) 3D reconstructed brain with EspINA analyzer, taking the same stack of MR images as the basis; (**O**) 3D reconstruction of the structures of interest, using EspINA software analyzer. Observe the orthogonal planes of the MR images after removing the neocortex; (**P**) same as in (**O**) without the orthogonal planes. Scale bar in (**L**): 20 mm in (**A**–**L**).

**Figure 3:**
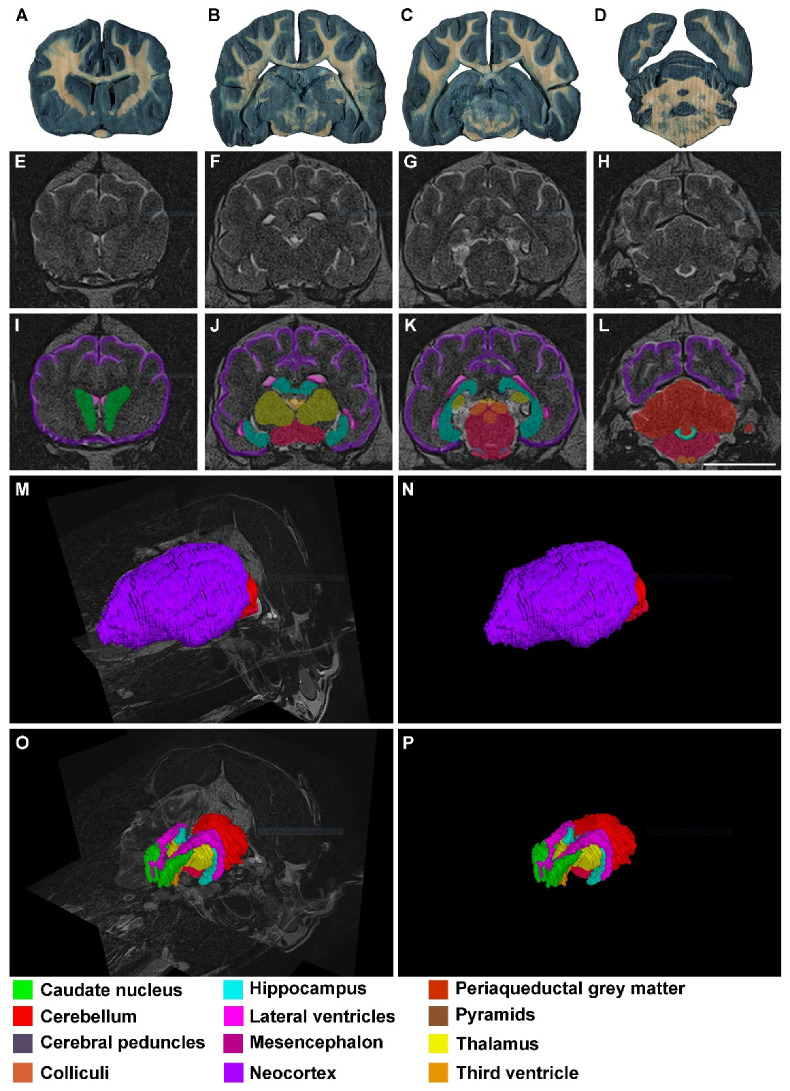
Images of dog brains without any signs of neural disease: (**A**–**D**) thick transversal sections (0.3 mm) stained with Mulligan’s solution. The levels are approximately equivalent to that shown in (**E**–**H**) and (**I**–**L**); (**E**–**H**) dog 1 in vivo transversal MR images (1.5 T) in the anterior–posterior axis; (**I**–**L**) segmented structures with EspINA analyzer (see the color legend at the bottom); (**M**) 3D reconstructed brain with ImageJ software, taking the same stack of MR images as the basis; (**N**) 3D reconstructed brain with EspINA analyzer, taking the same stack of MR images as the basis; (**O**) 3D reconstruction of the structures of interest, using EspINA analyzer. Observe the orthogonal planes of the MR images after removing the neocortex; (**P**) same as in (**O**) without the orthogonal planes. Scale bar in (**L**): 20 mm in (**A**–**L**).

**Figure 4:**
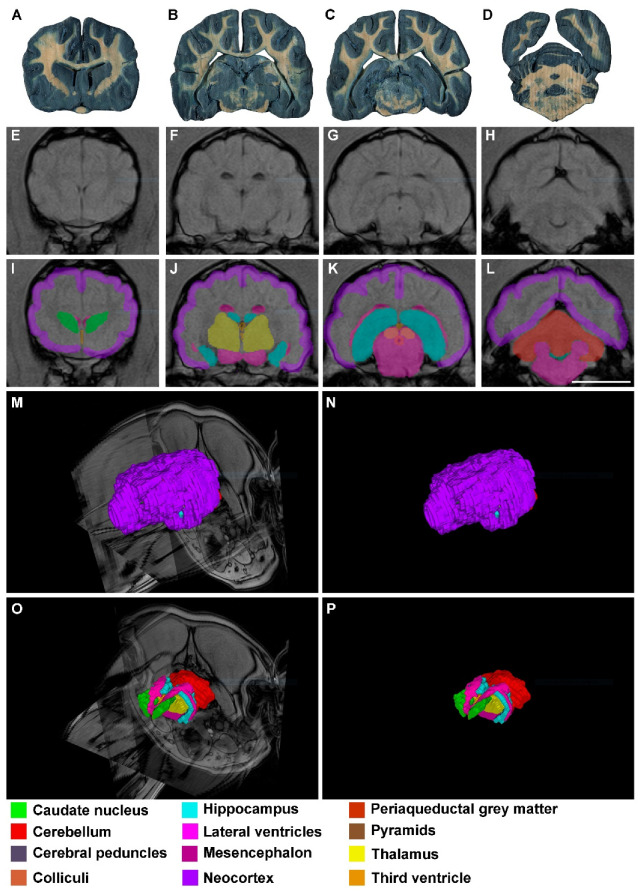
Images of dog brains without any sign of neural disease: (**A**–**D**) thick transversal sections (0.3 mm) stained with Mulligan’s solution. The levels are approximately equivalent to that shown in (**E**–**H**) and (**I**–**L**); (**E**–**H**) dog 3 in vivo transversal MR images (0.23 T) in the anterior–posterior axis; (**I**–**L**) segmented structures with EspINA analyzer (see the color legend at the bottom); (**M**) 3D reconstructed brain with ImageJ, taking the same stack of MR images as the basis; (**N**) 3D reconstructed brain with EspINA analyzer, taking the same stack of MR images as the basis; (**O**) 3D reconstruction of the structures of interest, using the EspINA software. Observe the orthogonal planes of the MR images after removing the neocortex; (**P**) same as in (**O**) without the orthogonal planes. Scale bar in (**L**): 20 mm in (**A**–**L**).

As mentioned before, segmentations and reconstructions were possible in all cases, even in those from low resolution (0.23 T) MR images. Therefore, the quality of the results from 4.7 T, 1.5 T and 0.23 T MR images was good enough for students to recognize these structures and to understand the topographical relation they have (Figure 2, Figure 3 and Figure 4).

**Figure 5:**
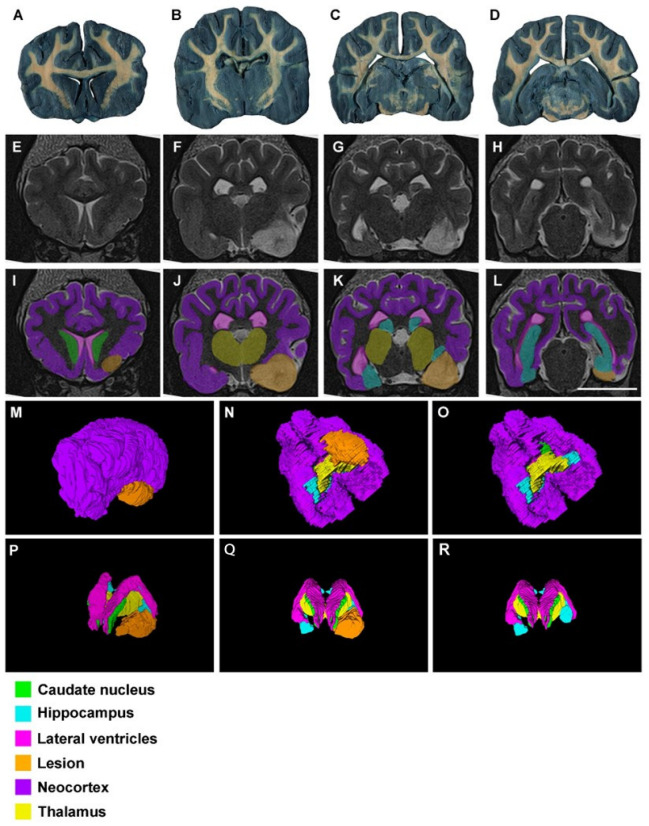
Images of a normal mixed-breed dog brain (**A**–**D**) and a 9 year old French bulldog with a tumor (dog 2) (**E**–**R**): (**A**–**D**) thick transversal sections (0.3 mm) stained with Mulligan’s solution. There are no signs of neural disease. The levels are approximately equivalent to that shown in (**E**–**H**) and (**I**–**L**); (**E**–**H**) in vivo transversal MR images (1.5 T) in the anterior–posterior axis. Note the presence of a lesion located ventrally, extending from the caudate nucleus level rostrally to the mesencephalon caudally; (**I**–**L**) EspINA segmented structures of interest in relation to the lesion (in orange) location (see the color legend at the bottom); (**M**) craniolateral EspINA 3D reconstructed brain. Observe the lesion in orange; (**N**) ventral EspINA 3D reconstructed brain. Observe the lesion in orange. (**O**) has the same 3D view as in (**N**). This time, the lesion has been removed to show the possibilities of our innovative tool; (**P**,**Q**) craniolateral and cranial 3D brain reconstructions, respectively. Observe the lesion (in orange) surrounded by some of the structures of interest, after removing the neocortex; (**R**) is the same as in (**Q**), after the lesion removal to show that, among other brain regions, the lesion affects the pyriform lobe and the hippocampus and compresses the lateral ventricle of the left hemisphere. Scale bar in (**L**): 20 mm in (**A**–**L**).

**Figure 6:**
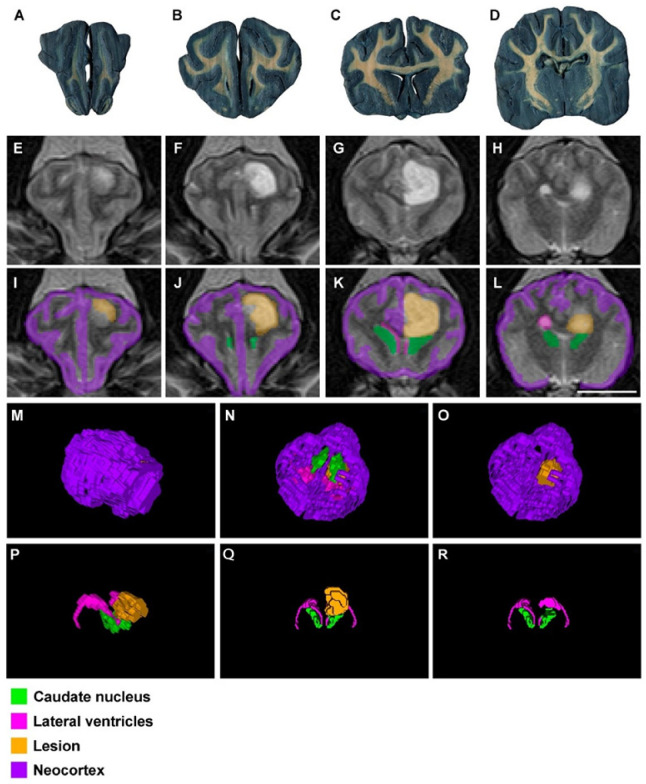
Images of a normal mixed-breed dog brain (**A**–**D**) and a 6 year old French bulldog with a tumor (dog 4) (**E**–**R**): (**A**–**D**) thick transversal sections (0.3 mm), stained with Mulligan’s solution. There are no signs of neural disease. The levels are approximately equivalent to that shown in (**E**–**H**) and (**I**–**L**); (**E**–**H**) in vivo transversal MR images (0.23 T) in the anterior–posterior axis. Note the presence of a glioma located dorsally, extending from the rostral part of the telencephalon, dorsal to the caudate nucleus, to the diencephalon caudally; (**I**–**L**) EspINA segmented structures of interest, in relation to the lesion (in orange) location (see the color legend at the bottom); (**M**) dorsolateral 3D view of the EspINA reconstructed brain. Observe the lesion (barely visible) in orange; (**N**) ventral EspINA reconstructed brain. Observe the lesion in orange and its relation to different neural structures. (**O**) has the same 3D view as in (**N**). This time, the neural structures have been removed to show the possibilities of our innovative tool; (**P**,**Q**) lateral and cranial 3D EspINA reconstructions, respectively. Observe the lesion (in orange) surrounded by some of the structures of interest, after removing the neocortex; (**R**) is the same as in (**Q**), after the lesion removal to show that, among other brain regions, the lesion affects the sensorimotor cortex, the caudate nucleus and compressed lateral ventricle of the right hemisphere. Scale bar in (**L**): 20 mm in (**A**–**L**).

To demonstrate the value of our teaching tool, we segmented and reconstructed in 3D 2 similar clinical cases, from different MR images 1.5 T and 0.23 T. The first case is shown in Figure 5. A French bulldog (dog 2) revealed the presence of an intra-axial and rounded mass (16 × 16 × 22 mm) localized in the left piriform lobe, by means of the 1.5 T MRI scanner. The lesion was T2W and Fluid-attenuated Inversion Recovery (FLAIR) hyperintense and T1-weighted (T1W) hypointense and showed a mild peripheral contrast enhancement that produced a mild peripheral vasogenic edema and a discrete mass effect, produced by the lesion and the edema itself. This lesion could be compatible with a neoplastic process, most likely a low-grade glioma such as an astrocytoma, due to the location, appearance and breed predisposition. The study also showed the presence of a left-sided otitis media, likely secretory, and aberrant turbinates, as an additional finding. Figure 6 shows images of another French bulldog (dog 4) that was scanned with the 0.23 T low field MRI. The encephalon presented an intra-axial oval mass (17.4 × 15.8 × 13.2 mm) located in the left-brain hemisphere. The lesion was hyperintense in T2W and FLAIR, and hypointense and mildly heterogeneous in T1W and T2*W images. No peripheral edema or cerebellar herniation were identified. In addition, a dorsolateral band of contrast enhancement was evident in post-contrast T1W images. The white matter and the corpus callosum appeared partially obliterated by the mass. On this occasion, a glioma was also considered for the main differential diagnosis, although other neoplastic or inflammatory conditions could not be ruled out. As shown in Figure 5 and Figure 6, in both cases it was possible to segment and reconstruct the lesion as well as the surrounding structures from MR images of different resolutions. Thus, for the first time, our results reveal that EspINA is a very suitable analyzer to create and combine this type of 3D reconstructions, including pathological cases.

**Figure 7:**
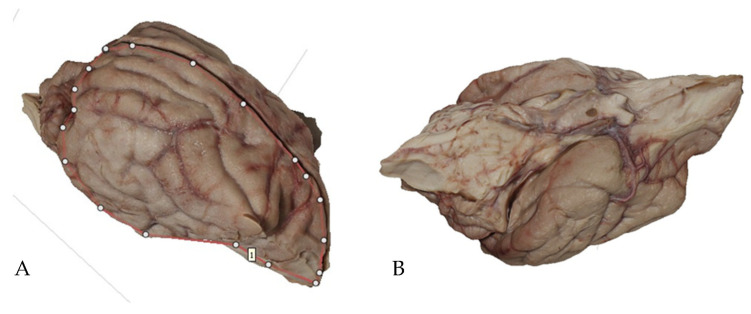
3D models of the whole brain reconstructed by means of photogrammetry. (**A**) dorsolateral view; (**B**) ventral view.

Concerning photogrammetry, it was possible to generate very precise 3D models from the whole brain (Figure 7). However, it was not possible to reconstruct either brain sections or MR images with enough quality to be used as a teaching tool.

## 4. Discussion

As mentioned before, the main goal of this present paper was to ascertain the value of EspINA to generate neural 3D reconstructions, proceeding from different quality MR images (4.7 T, 1.5 T and 0.23 T) and thus, to create a useful tool not only for anatomy students, but additionally for clinicians to understand and identify a multitude of neurological problems. An additional advantage of our educational tool is the possibility to combine neuroanatomy and clinical neurology. According to our experience, students frequently face many difficulties in their learning of anatomy and particularly of neuroanatomy. Hence, there have been continued efforts directed at incorporating new methods of teaching, learning and assessment that are aimed at logical learning and a long-term retention of anatomical knowledge. Thus, no single method for teaching anatomy, either medical or veterinary, can provide supremacy over another. On the other hand, anatomy study should not be completed in the first years of veterinary or medical school. Instead, students may continue the process of understanding anatomy (in the case of this present manuscript, neuroanatomy) in the higher years. To enhance this learning, it is normal to implement a type of “multimodal” system by combining different teaching systems: the more traditional approaches, with lectures and dissection combined with more innovative methods such as models, imaging, computer-assisted learning, peer teaching, 3D reconstructions or team-based learning [7,14,22,23,24,25,26,27,28,29,30,31,32,33].

Concerning medical neuroanatomy, different educational tools have been additionally developed over time [17,22,34,35,36]. In recent years, artificial intelligence (AI) has gained popularity. AI uses machine learning models to store, compute, analyze and even augment huge amounts of data to be retrieved when needed [37]. However, in our opinion, there are important limitations; for example, the cost of servers, training of students and employment of trained technical personnel to run and maintain either AI installations and apps or the development of tools. Furthermore, of course, this includes the need of adequate computers or internet connectivity to access such tools.

In summary, all these works have different approaches to the study of neuroanatomy and are, in general, useful educational tools that make the teaching–learning process much easier. However, it is noteworthy that, contrary to the method we propose with EspINA, in no case is anatomy combined with clinical neurology, which would be a further step in the integration of basic and applied disciplines, which should be one of the main goals of university education.

In respect to veterinary anatomy, the situation is very similar to that described before. The alternative methods to improve its learning, in comparison to other traditional techniques, are diverse [4,5,12,38,39,40,41,42,43,44,45,46,47,48,49,50]. If we focus on veterinary neuroanatomy, the use of digital teaching, 3D reconstructions and computer-assisted resources is also widespread [2,3,15,16]. Especially interesting is the use of MRI and CT scanning to create a fully interactive educational and training package for the neurovascular system of the canine brain [17]. They use a 1.5 T unit for the MR, Amira 5.6 for surface reconstruction and Mudbox to determine the aspect of the geometry. The authors combine automatic and manual segmentations, although they recognize that the poor signal from MR images 1.5 T may hinder the accurate reconstruction of anatomical structures, especially in the case of automatic segmentations. In addition, the MR dataset failed to provide an image of the entire brain. This means that the 3D reconstruction of these missing regions was by approximation, which could have impacted the anatomical accuracy of the canine brain. We consider this fact to be an important limitation, as accuracy is important when talking about neuroanatomy. In our case, it is possible to segment different quality MR images with EspINA, with high precision. Additionally, in our case, segmenting manually makes EspINA’s accuracy greater, taking into consideration that details are critical in anatomy to understand morphology, topography and the relations between brain structures.

Each of the methods described before offer their own advantages and disadvantages. An ideal example of our 3D EspINA reconstructions’ benefits is the possibility to focus on challenging complicated anatomical areas, or on different neural problems by segmenting the cerebral areas or structures of interest from different quality MR images (0.23, 1.5 and 4.7 T). For the first time, we have created an educational tool that combines neuroanatomy and neuropathology, contrary to what is reflected in previous publications, that are only focused on the brain anatomy. Despite all the potential benefits of this educational method, there are also a few limitations. For example, appreciating complex 3D spatial relationships requires a strong understanding of anatomy and mental 3D visualization skills, that not all students have. Another probable restriction is the use of manual segmentation tools, because to date, there are no automatic algorithms to adequately represent neural structures. Thus, using a more semi-automated or automated approach to the segmentation process could help to overcome this issue which, on the other hand, is possible when using EspINA to segment other brain structures, for example, different types of synapses [51]. In any case, we show this analyzer to be achievable in the creation of a training, educational and clinical application, using MR images of different qualities as the basis.

Although it is not the main goal of this present paper, we tried to determine if alternative methods used to make 3D reconstructions in other sciences could be applied to the study of the brain. As mentioned before, we tried to demonstrate whether photogrammetry could additionally be useful to produce 3D reconstructions of the brain, either from biological specimens, brain sections or MR images. At this point, there are no previous data in the literature on the use of photogrammetry in biology or veterinary medicine. Although our results so far are promising, we need further investigations to confirm if this is possible, as well as to discover the advantages and disadvantages in the use of this technique for the study of the encephalon.

## 5. Conclusions

Our contribution is important as we demonstrate, for the first time, that by using EspINA it is possible to obtain segmented structures and 3D reconstructions from 0.23, 1.5 and 4.7 T MR images. We show that this combination (imaging techniques and EspINA 3D reconstructions) is an optimal tool to help anatomists, veterinary students and clinicians have a better understanding of brain anatomy or neural lesion location. In other words, it is a step forward to facilitate the study and comprehension of neural structures for anatomy students and a new way for clinicians to accurately identify different lesions or pathologies, taking anatomy as the basis. Of course, this combination may not replace other methods to study brain anatomy; however, it can change the concept of veterinary anatomy teaching and learning. Our method provides a 3D reality that ensures the knowledge of neuroanatomy and can be a good basis for neurology practice, either medical or surgical. The lack of similar resources in this field suggests that this method introduces a new dimension into veterinary education and training. In summary, the use of EspINA 3D reconstructions is a step forward to integrate anatomy and clinical knowledge, as it has the potential to be used alongside other methodologies for the training of future veterinary practitioners.

On the other hand, we have investigated whether the use of photogrammetry could also be interesting in neuroanatomy 3D reconstructions. Our results are, so far, promising, especially when it comes to the reconstruction of the whole brain. However, it is still too early to confirm that photogrammetry is an additional interesting tool for teaching neuroanatomy.

## Data Availability

The data presented in this study are available on request from the corresponding author.

## References

[1] Wakuri H., Zhang Y., Liu B., Yang W. (1998). What is anatomy for? Considerations on the body as a whole and whole anatomy. Okajimas Folia Anat. Jpn..

[2] Schoenfeld-Tacher R.M., Horn T.J., Scheviak T.A., Royal K.D., Hudson L.C. (2017). Evaluation of 3D additively manufactured canine brain models for teaching veterinary neuroanatomy. J. Vet. Med. Educ..

[3] Foss K.D., Seals C.D.A., Hague D.W., Mitek A.E. (2022). Effectiveness of supplementary materials in teaching the veterinary neurologic Examination. J. Vet. Med. Educ..

[4] Khayruddeen L., Livingstone D., Ferguson E. (2019). Creating a 3D learning tool for the growth and development of the craniofacial skeleton. Adv. Exp. Med. Biol..

[5] Guimarães B., Firmino-Machado J., Tsisar S., Viana B., Pinto-Sousa M., Vieira-Marques P., Cruz-Correia R., Ferreira M.A. (2019). The role of anatomy computer-assisted learning on spatial abilities of Medical students. Anat. Sci. Educ..

[6] Memon I. (2018). Cadaver dissection is obsolete in medical training! A misinterpreted notion. Med. Princ. Pract..

[7] Araujo Cuauro J.C. (2018). Aspectos históricos de la enseñanza de la anatomía humana desde la época primitiva hasta el siglo XXI en el desarrollo de las ciencias morfológicas. Rev. Argent. Anat. Online.

[8] Van Ginnekeng C.J., Vanthournout G. (2005). Rethinking the learning and evaluation environment of a veterinary course in gross anatomy: The implementation of an assessment and development center and an E-Learning platform. J. Vet. Med. Educ..

[9] Guevar J. (2020). The evolution of educational technology in veterinary anatomy education. Adv. Exp. Med. Biol..

[10] Krucker T., Lang A., Meyer E.P. (2006). New polyurethane-based material for vascular corrosion casting with improved physical and imaging characteristics. Microsc. Res. Tech..

[11] Riederer B.M. (2014). Plastination and its importance in teaching anatomy. Critical points for long-term preservation of human tissue. J. Anat..

[12] Croy B.A., Dobson H. (2003). Radiology as a tool for teaching veterinary anatomy. J. Vet. Med. Educ..

[13] Sandhu G.S., Solorio L., Broome A.M., Salem N., Kolthammer J., Shah T., Flask C., Duerk J.L. (2010). Whole animal imaging. Rev. Syst. Biol. Med..

[14] Langlois J., Bellemare C., Toulouse J., Wells G.A. (2020). Spatial abilities training in anatomy education: A systematic review. Anat. Sci. Educ..

[15] Christ R., Guevar J., Poyade M., Rea P.M. (2018). Proof of concept of a workflow methodology for the creation of basic canine head anatomy veterinary education tool using augmented reality. PLoS ONE.

[16] Hadžiomerović N., Hadžiomerović A.I., Avdić R., Muminović A., Tandir F., Bejdić P., Pandžić A. (2023). Students’ performance in teaching neuroanatomy using traditional and technology-based methods. Anat. Histol. Embryol..

[17] Raffan H., Guevar I., Poyade M., Rea P.M. (2017). Canine neuroanatomy: Development of a 3D reconstruction and interactive ap-plication for. PLoS ONE.

[18] Falkingham P.L. (2012). Acquisition of high-resolution three-dimensional models using free, open-source, photogrammetric software. Palaeontol. Electron..

[19] Herrero M.J., Pérez-Fortes A.P., Escavy J.I., Insua-Arévalo J.M., De La Horra R., López-Acevedo F., Trigos L. (2022). 3D model generated from UAV photogrammetry and semi-automated rock mass characterization. Comput. Geosci..

[20] Forlizzi V., Miranda-Solís F., Pérez Cruz J.C., Cahuantico-Choquevilca L.A., Morán G., Baldoncini M. (2020). Tinción de Mulligan en Neuroanatomía: Protocolización de la técnica. Rev. Argent. Anat. Online.

[21] Bythell J., Pan P., Lee J. (2001). Three-dimensional morphometric measurements of reef corals using underwater photogrammetry techniques. Coral Reefs.

[22] Krontiris-Litowitz J. (2008). Using truncated lectures, conceptual exercises, and manipulatives to improve learning in the neuroanatomy classroom. Adv. Physiol. Educ..

[23] Petersson H., Sinkvist D., Wang C., Smedby O. (2009). Web-based interactive 3D visualization as a tool for improved anatomy learning. Anat. Sci. Ed..

[24] Johnson E.O., Charchanti A.V., Troupis T.G. (2012). Modernization of an anatomy class: From conceptualization to implementation. A case for integrated multimodal–multidisciplinary teaching. Anat. Sci. Educ..

[25] Preece D., Williams S.B., Lam R., Weller R. (2013). “Let’s get physical”: Advantages of a physical model over 3D computer models and textbooks in learning imaging anatomy. Anat. Sci. Educ..

[26] Ghosh S.K. (2017). Cadaveric dissection as an educational tool for anatomical sciences in the 21st century. Anat. Sci. Educ..

[27] Langlois J., Bellemare C., Toulouse J., Wells G.A. (2017). Spatial abilities and anatomy knowledge assessment: A systematic review. Anat. Sci. Educ..

[28] Joseph M., Singh B. (2019). Recent advances and changing face of anatomy teaching and Learning in medical education. Natl. J. Clin. Anat..

[29] Yuen J. (2020). What is the role of 3D printing in undergraduate anatomy education? A scoping review of current literature and recommendations. Med. Sci. Educ..

[30] Bernard F., Richard P., Kahn A., Fournier H.D. (2020). Does 3D stereoscopy support anatomical education?. Surg. Radiol. Anat..

[31] Bogomolova K., Vorstenbosch M.A.T.M., El Messaoudi I., Holla M., Hovius S.E.R., van der Hage J.A., Hierck B.P. (2023). Effect of binocular disparity on learning anatomy with stereoscopic augmented reality visualization: A double center randomized controlled trial. Anat. Sci. Educ..

[32] Chytas D., Salmas M., Demesticha T., Noussios G., Paraskevas G., Chrysanthou C., Asouhidou I., Katsourakis A., Fiska A. (2021). A review of the use of virtual reality for teaching radiology in conjunction with anatomy. Cureus.

[33] Asghar A., Naaz S., Patra A., Ravi K.S., Khanal L. (2022). Effectiveness of 3D-printed models prepared from radiological data for anatomy education: A meta-analysis and trial sequential analysis of 22 randomized, controlled, crossover trials. J. Educ. Health Promot..

[34] Manson A., Poyade M., Rea P. (2015). A recommended workflow methodology in the creation of an educational and training application incorporating a digital reconstruction of the cerebral ventricular system and cerebrospinal fluid circulation to aid anatomical understanding. BMC Med. Imaging.

[35] Ocampo-Navia M.I., Gómez-Vega J.C. (2021). Techniques for an anatomic approach to the study of the brain: A review. Univ. Med..

[36] Jacquesson T., Simon E., Dauleac C., Margueron L., Robinson P., Mertens P. (2020). Stereoscopic three-dimensional visualization: Interest for neuroanatomy teaching in medical school. Surg. Radiol. Anat..

[37] Abdellatif H., Al Mushaiqri M., Albalushi H., Al-Zaabi A.A., Roychoudhury S., Das S. (2022). Teaching, learning and assessing anatomy with artificial intelligence: The road to a better future. Int. J. Environ. Res. Public Health.

[38] Leandro R.M., Foz Filho R.P.P., De Silvio M.M., Quilici A.P., Sattin M.M., Paretsis B.F., Souza V.A. (2019). Construction of the equine digestive system: A tool for teaching topographical anatomy. J. Vet. Med. Educ..

[39] Xiberta P., Boada I. (2019). IVET, an interactive veterinary education tool. J. Anim. Sci..

[40] Suñol A., Aige V., Morales C., López-Beltran M., Feliu-Pascual A.L., Puig J. (2019). Use of three-dimensional printing models for veterinary medical education: Impact on learning how to identify canine vertebral fractures. J. Vet. Med. Educ..

[41] Little W.B., Artemiou E., Fuentealba C., Conan A., Sparks C. (2019). Veterinary students and faculty partner in developing a virtual three-dimensional (3D) interactive touch screen canine anatomy table. Med. Sci. Educ..

[42] Wilhite R., Wölfel I. (2019). 3D Printing for veterinary anatomy: An overview. Anat. Histol. Embryol..

[43] DeBose K. (2020). Virtual anatomy: Expanding veterinary student learning. J. Med. Libr. Assoc..

[44] Oberoi G., Eberspächer-Schweda M.C., Hatamikia S., Königshofer M., Baumgartner D., Kramer A.M., Schaffarich P., Agis H., Moscato F., Unger E. (2020). 3D printed biomimetic rabbit airway simulation model for nasotracheal intubation training. Front. Vet. Sci..

[45] Lee J.S., Park T.H., Ryu J.Y., Kim D.K., Oh E.J., Kim H.M., Shim J.-H., Yun W.-S., Huh J.B., Moon S.H. (2021). Osteogenesis of 3D-printed PCL/TCP/bdECM scaffold using adipose-derived stem cells aggregates; an experimental study in the canine mandible. Int. J. Mol. Sci..

[46] Canright A., Bescoby S., Dickson J. (2023). Evaluation of a 3D computer model of the equine paranasal sinuses as a tool for veterinary anatomy education. J. Vet. Med. Educ..

[47] Plewa B., Skieresz-Szewczyk K., Jackowiak H. (2022). Three-dimensional characteristic of fungiform papillae and its taste buds in European bison (*Bison bonasus*), cattle (*Bos taurus*), and Bison bonasus hybrid. BMC Vet. Res..

[48] Senos R., Ramos Leite C.A., Dos Santos Tolezano F., Roberto-Rodrigues M., Pérez W. (2023). Using videos in active learning: An experience in veterinary anatomy. Anat. Histol. Embryol..

[49] Kapoor K., Singh A. (2022). Veterinary anatomy teaching from real to virtual reality: An unprecedented shift during COVID-19 in socially distant era. Anat. Histol. Embryol..

[50] Hammerton C., Yip S.W.L., Manobharath N., Myers G., Sturrock A. (2022). Are 3D printed models acceptable in assessment?. Clin. Teach..

[51] Montero-Crespo M., Dominguez-Alvaro M., Rondon-Carrillo P., Alonso-Nanclares L., DeFelipe J., Blazquez-Llorca L. (2020). Three-dimensional synaptic organization of the human hippocampal CA1 field. Elife.

